# A reinforcement learning model with choice traces for a progressive ratio schedule

**DOI:** 10.3389/fnbeh.2023.1302842

**Published:** 2024-01-10

**Authors:** Keiko Ihara, Yu Shikano, Sae Kato, Sho Yagishita, Kenji F. Tanaka, Norio Takata

**Affiliations:** ^1^Division of Brain Sciences, Institute for Advanced Medical Research, Keio University School of Medicine, Tokyo, Japan; ^2^Department of Biology, Stanford University, Stanford, CA, United States; ^3^Center for Disease Biology and Integrative Medicine, Faculty of Medicine, The University of Tokyo, Tokyo, Japan

**Keywords:** choice stickiness, dopamine, fiber photometry, methamphetamine, mouse, operant conditioning, reward prediction error, ventral striatum

## Abstract

The progressive ratio (PR) lever-press task serves as a benchmark for assessing goal-oriented motivation. However, a well-recognized limitation of the PR task is that only a single data point, known as the breakpoint, is obtained from an entire session as a barometer of motivation. Because the breakpoint is defined as the final ratio of responses achieved in a PR session, variations in choice behavior during the PR task cannot be captured. We addressed this limitation by constructing four reinforcement learning models: a simple Q-learning model, an asymmetric model with two learning rates, a perseverance model with choice traces, and a perseverance model without learning. These models incorporated three behavioral choices: reinforced and non-reinforced lever presses and void magazine nosepokes, because we noticed that male mice performed frequent magazine nosepokes during PR tasks. The best model was the perseverance model, which predicted a gradual reduction in amplitudes of reward prediction errors (RPEs) upon void magazine nosepokes. We confirmed the prediction experimentally with fiber photometry of extracellular dopamine (DA) dynamics in the ventral striatum of male mice using a fluorescent protein (genetically encoded GPCR activation-based DA sensor: GRAB_DA2m_). We verified application of the model by acute intraperitoneal injection of low-dose methamphetamine (METH) before a PR task, which increased the frequency of magazine nosepokes during the PR session without changing the breakpoint. The perseverance model captured behavioral modulation as a result of increased initial action values, which are customarily set to zero and disregarded in reinforcement learning analysis. Our findings suggest that the perseverance model reveals the effects of psychoactive drugs on choice behaviors during PR tasks.

## Introduction

A progressive ratio (PR) schedule in reinforcement learning (RL) is a popular task to measure reward strength (Hodos, 1961; Richardson and Roberts, 1996) and behavioral motivation (Tsutsui-Kimura et al., 2017b; Zhou et al., 2022), but its deficiencies have been well recognized for years (Richardson and Roberts, 1996; Chen et al., 2022). The most significant limitation is that a stream of choice behaviors during the PR session, which commonly takes an hour or more, is discarded, and only a single data point, a breakpoint, is provided from an entire session of a PR task (Arnold and Roberts, 1997). In a PR schedule, response requirements to earn a reward escalate after delivery of each reinforcement, e.g., the number of lever presses required to obtain a single reward increases from 1, 2, 4, … along with trials (Richardson and Roberts, 1996). The highest number of lever presses achieved in a PR session is defined as the breakpoint and is used as a barometer of motivation (Chen et al., 2022). Although modulation of breakpoints by psychostimulants has been used to investigate the effects of these drugs (Thompson, 1972), variations in choice behavior during the PR task cannot be captured. Therefore, a method to assess the choice behavior may enable exploration of novel effects of psychostimulants.

Methamphetamine (METH) is a psychoactive dopaminergic drug with a wide variety of effects, including motivational and behavioral effects. Low-dose METH may modulate choice behavior during PR tasks, and this cannot be captured by a breakpoint. Indeed, METH induces qualitatively different effects as a function of dose (Grilly and Loveland, 2001). Moderate doses (1.0–2.0 mg/kg) of METH increase PR task breakpoints (Thompson, 1972; Bailey et al., 2015), but low doses (0.3–0.6 mg/kg) of METH have not been reported to exert such modulation (Grilly and Loveland, 2001; Shen et al., 2010). Still, low-dose METH has many other psychological and behavioral effects, including performance improvements in reversal learning (Kulig and Calhoun, 1972; Calhoun and Jones, 1974) and induction of behavioral activation (Hall et al., 2008; Miller et al., 2013) (but see Asami and Kuribara, 1989; Jing et al., 2014). Clinical application of low-concentration dopaminergic drugs for severe post-traumatic stress disorder (PTSD) (Mithoefer et al., 2019) and attention deficit hyperactivity disorder (ADHD) (Guo et al., 2023) further underlines the necessity of developing a quantitative method to enable analysis of behavioral effects by low-dose METH.

Reinforcement learning (RL) algorithms are used to construct normative models that generate subsequent choice behavior based on a history of behavioral selections and rewards (Niv et al., 2007; Niv, 2009). RL models make it possible to relate computation and neurophysiological dynamics, such as encoding of reward prediction error (RPE) by the extracellular dopamine (DA) concentration in the stratum (Schultz et al., 1997). We constructed an RL model for a fixed ratio (FR), lever-press task for mice (Shikano et al., 2023). In the FR schedule, response requirements to earn a reward are fixed (Yokel and Wise, 1975). In our study, we used FR5 tasks that required mice to press a lever five times for a reward. To model mouse behavior during FR5 tasks, we constructed an RL model that had multiple state values. Each state value corresponded to a lever-press number, e.g., a state value *V*_2_ represents a state in which mice pressed a lever twice. Multiple state values in the model assume that mice have an internal representation for each lever-press number. It is unlikely, however, that mice possess an internal representation for each lever press in the case of a PR schedule because the number of lever presses for a reward increases, rapidly exceeding 100. This difficulty may be one of reasons that RL models for PR tasks have apparently not been proposed. A situation in which numerous lever presses are required for mice to obtain a single reward during the latter half of PR tasks resembles a sparse reward environment. A recent study proposed that asymmetric learning rates are necessary for an RL model that describes persistent choice behavior of mice in a scarce-reward environment, where the probability for obtaining a reward is small (Ohta et al., 2021). In that study, a large learning rate for a positive RPE, i.e., obtaining a reward, and a small learning rate for a negative RPE, i.e., an unexpected omission of a reward, were proposed as a mechanism for exerting a behavior repeatedly without a reward. Another study, however, demonstrated theoretically that persistent lever-pressing behavior is described by an RL model with a choice trace rather than asymmetric learning rates (Katahira, 2015, 2018; Sugawara and Katahira, 2021). It is not clear which model, an asymmetric learning rate model or a choice-trace model, better describes choice behavior during PR lever-press tasks.

In this study, we propose a RL model with choice traces to realize analysis of choice behavior during PR lever-press tasks. We combined a PR lever-press task in mice, computational modeling of the behavior, and DA measurements in the ventral striatum (VS) of mice. We found that PR lever-press tasks for mice can be described as a three-choice behavior, rather than two, because mice performed numerous magazine nosepokes to check a food reward, in addition to conventional active and inactive lever presses. A Q-learning model with choice traces was the best-fitting model as it predicted gradual modulation of RPEs during PR tasks. We confirmed the prediction with DA measurements during the PR tasks by mice. We applied the perseverance model to experiments with low-dose METH, which did not change breakpoints, but increased magazine nosepokes during a PR session. The higher frequency of magazine nosepokes during PR tasks was described as increases of initial action values. The perseverance model realizes examination of choice behavior in PR tasks, which helps to describe the effects of psychiatric drugs using PR tasks.

## Materials and methods

### Animals

All animal experiments were approved by the Animal Ethics Committee of Keio University, Japan (approval A2022-301). Eleven 3 months-old male C57BL/6 mice weighing 23–27 g, purchased from SLC (Shizuoka, Japan), were used. Male mice were used because it is reported that estrous cycle affects performance in PR tasks in rodents (Roberts et al., 1989) and that gender differences exist in behavioral effects of METH, including PR schedules (Schindler et al., 2002; Roth and Carroll, 2004). Mice were housed individually and maintained on a 12 h light/12 h dark schedule, with lights off at 8:00 PM. Their body weights were maintained at 85% of their initial body weight under conditions of food restriction with water *ad libitum*.

### Surgery

Mice were anesthetized by intraperitoneal injection of ketamine (100 mg/kg) and xylazine (10 mg/kg) before a stereotaxic surgery for adeno-associated virus (AAV) injection and implantation of an optic fiber that targeted the right VS (Supplementary Figure S3). Details for surgical procedures were described in detail previously (Shikano et al., 2023). In brief, following an incision in the scalp, a craniotomy with a diameter of 1.5 mm was created above the right VS at stereotaxic coordinates 1.1 mm anteroposterior (AP) and 1.9 mm mediolateral (ML) to the bregma. The dura mater was surgically removed. A total volume of 0.5 μL GRAB_DA2m_ virus (PHP.eB AAV-hSyn-GRAB-DA2m-W, 1.0 × 10^14^ genome copies/mL) (Sun et al., 2018, 2020) was injected with a pulled glass micropipette into the VS (3.5 to 3.7 mm dorsoventral (DV) relative to the cortical dura surface) according to the atlas of Franklin and Paxinos (2008). The injection was driven at a 100 nL/min flow rate by a microinjector (Nanoliter 2020 Injector, World Precision Instruments, Sarasota, FL). The micropipette was left in place for another 5 min to allow for tissue diffusion before being retracted slowly. Following the GRAB_DA2m_ virus injection, an optical fiber cannula (CFMC14L05, 400 μm in diameter, 0.39 NA; Thorlabs, Newton, NJ) attached to a ceramic ferrule (CF440-10, Thorlabs) and a ferrule mating sleeve (ADAF1-5, Thorlabs) was inserted into the same side of the VS as the virus injection and cemented in place (3.4 to 3.6 mm DV). Operant conditioning and data collection were started more than 4 days after the surgery to allow the mice time to recover. As we reported previously, optical fiber implantation in the ventral striatum does not modify the lever-press behavior of mice (Extended Data Figure 2-1C in Shikano et al., 2023).

### Behavioral task

**Figure 1:**
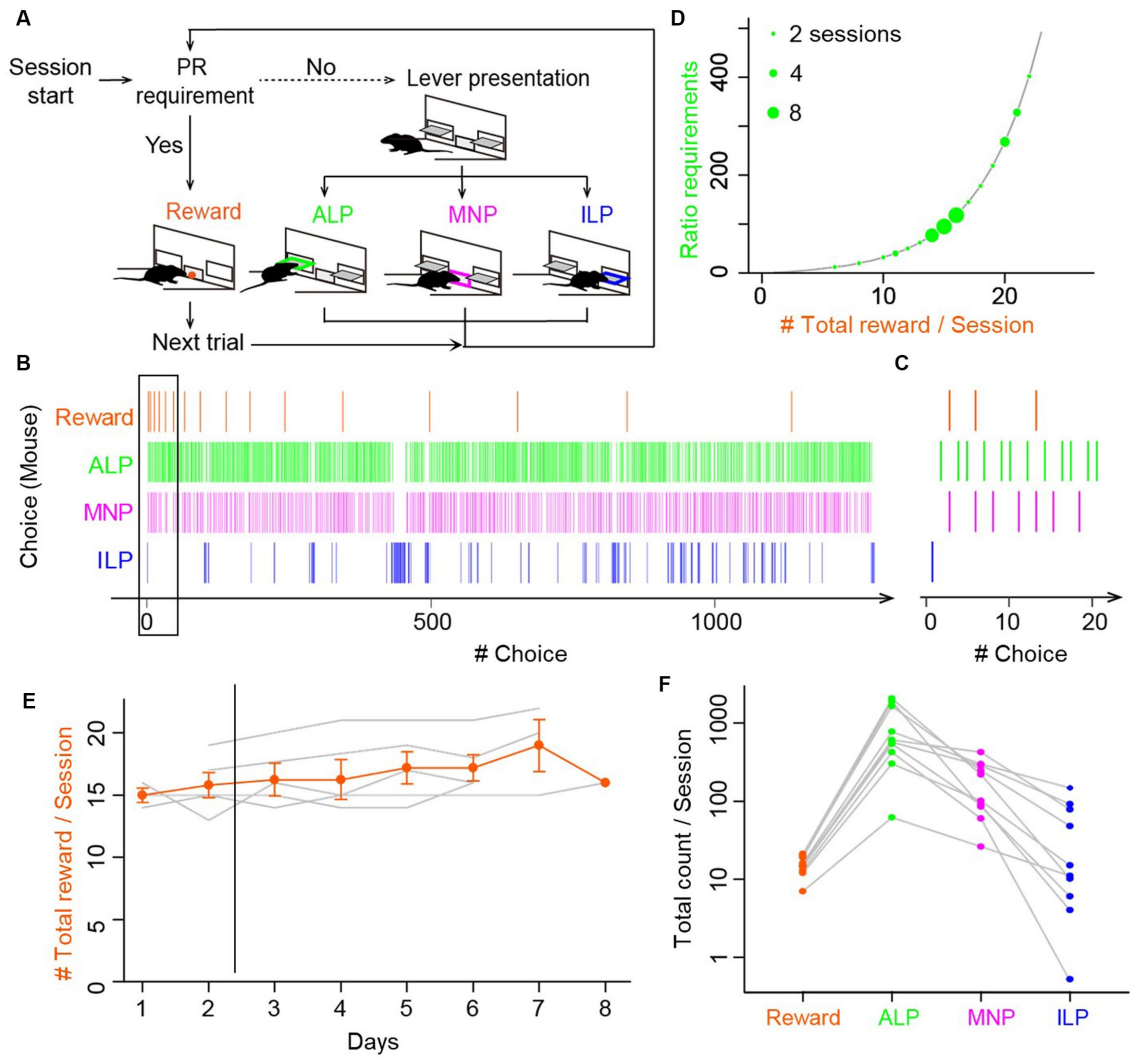
Mice performed void magazine nosepokes as frequently as active and inactive lever presses during progressive ratio lever-press tasks. **(A)** Choice behavior by mice during the progressive ratio (PR) lever-press task. A session started with presentation of active and inactive levers (lever presentation). Mice performed either an active lever press (ALP), a magazine nokepoke (MNP), or an inactive lever press (ILP). If mice pressed an active lever a certain number of times (PR requirement), a food reward was delivered at the magazine (reward), followed by a subsequent trial after an inter-trial interval of 30 s. The number of ALPs required to obtain a reward increased progressively in every trial. **(B)** A representative time course of a choice behavior by a mouse in a whole session of a PR task. The session was terminated by a 60 min time limit, during which the mouse earned 17 food rewards. Intervals of the food rewards (orange) increased due to the exponential increase of the ratio requirement. The mouse checked a magazine without a reward (MNP, magenta) before completing the ratio, in addition to ALP and ILP. **(C)** Enlarged view of a time course of a choice behavior (black rectangle in **B**), showing the first four trials in the session in the PR lever-press task by the mouse. Magazine-checking behavior (magenta) occupied a non-negligible percentage in the choice behavior during the PR task. **(D)** The ratio requirement during a PR task. The number of ALP responses required to earn a food reward in a trial increased exponentially in the order: 1, 2, 4, 6, 9, 12, 15, 20, 25, 32, 40, 50, 62, 77, 95, and so on (black line). The *y*-axis corresponds to breakpoints, which are the final ratios completed in a session. Green circles show breakpoints by 13 mice in 38 sessions. Diameters of circles show the number of sessions by mice. **(E)** PR lever-press tasks were stable during days 3–8. Mice performed the PR task one session per day for 6 to 8 consecutive days. Total reward counts per session (breakpoints) deviated less than 15% after day 3, data of which were used for the following analysis and modeling. Gray lines show mean reward counts per session of each mouse. **(F)** Comparison of total counts of behavioral choices in a session of the PR task. The relative frequency was relatively consistent: ALP > MNP > ILP. Gray lines show data for each mouse. Circles denote average counts of multiple sessions for each mouse (*n* = 8 mice, 24 sessions).

Mice were food-restricted and trained to perform a lever-pressing operant conditioning task in FR and PR schedules to retrieve a food pellet, as described previously (Tsutsui-Kimura et al., 2017b; Shikano et al., 2023). Behavioral training and tests were performed under constant darkness in an aluminum operant chamber (21.6 × 17.6 × 14.0 cm, Med Associates, Fairfax, VT) housed within a sound-attenuating enclosure in a daytime. The chamber was equipped with two retractable levers (located 2 cm above the floor) and one food magazine between the levers on the floor (Figure 1A). Each trial began with extension of the levers. As for three mice (mouse ID: VLS06, VLS09, VLS10) of the 11, a 5 s sound cue (80 dB) from a speaker located on the opposite wall preceded the lever extension. Presses on the lever on the left of the food magazine (reinforced side) were counted (active lever press: ALP), and a reward pellet (20 mg each, Dustless Precision Pellets, Bio-Serv, Flemington, NJ) was dispensed to the magazine immediately after the required number of presses was made. The levers were retracted at the same time as the reward delivery. In contrast, presses on the other lever on the right side were counted, but had no programmed consequence (non-reinforced side; inactive lever press: ILP). A refractory period of 0.5 s followed pressing of a lever, either active or inactive, before the lever was re-extended. Note that (1) inter-press intervals of lever pressing in our PR tasks, i.e., ~0.9 s in our study were comparable to those of previous reports without the refractory period (Tsibulsky and Norman, 2022), and (2) the ratio of total magazine nosepokes (183) and active lever presses (885) during a PR session (Figure 1F) were similar too to those in a previous study without the refractory period (Ko and Wanat, 2016), implying that the refractory period does not have significant effects on the mouse behavior during these PR tasks. In addition to pressing the reinforced and non-reinforced levers, mice occasionally poked into the magazine (magazine nosepoke) before making the required number of lever presses (Ko and Wanat, 2016; Zhou et al., 2022; Shikano et al., 2023). The timing of a magazine nose poke was defined as the time point when the distance of the animal’s head to the center of the magazine became less than 2.5 cm (Supplementary Figure S4). An inter-trial interval (ITI) of 30 s (or 35 s in the presence of the sound cue) followed each food delivery, during which the levers were not presented, and mice consumed the reward. The subsequent trial was automatically initiated after the ITI period ended. TTL signals were generated at the timings of lever extension and lever pressing and digitized by a data acquisition module (cDAQ-9178, National Instruments, Austin, TX). TTL signals were simultaneously recorded at a sampling frequency of 1,000 Hz by a custom-made program (LabVIEW 2016, National Instruments) using voltage input modules (NI-9215, National Instruments). A single session for the operant conditioning task lasted for 60 min until the mice received 100 food rewards, or when the mice stayed away from the active lever for more than 5 min. To track the moment-to-moment position of the mice, an infrared video camera (ELP 2 Megapixel WEB Camera, OV2710, Ailipu Technology Co., Ltd., Shenzhen, China) was attached to the ceiling of the enclosure. Reflective tapes were attached to a custom-made 3D printed optical fiber protector (1.2 × 1.4 cm) on the head of the mice. The tapes were recorded at a sampling rate of 20 Hz. Mouse positions in each frame were computed offline with a custom-made code (MATLAB 2021a, MathWorks). The entire experimental procedure took 26–32 days, consisting of surgery, recovery, training in FR tasks, and test in PR tasks. Variation in the duration occurred because numbers of days required to recover from surgery and to complete the training differed among mice. Behavioral data were summarized as binary data with actions for active and inactive lever presses, magazine nosepokes, and a reward.

#### Fixed and progressive ratio tasks

FR sessions were used as a training of mice to associate lever pressing and a food reward. Mice were required to perform a fixed number of responses (lever presses) to attain a reward: one response was required in an FR1 schedule, and five consecutive responses were required in an FR5 schedule. Mice were trained for at least three sessions (one 60 min session/day) on the FR1 schedule followed by four sessions on the FR5 schedule. FR sessions were finished when the mice accomplished 100 completed trials or spent 60 min for a session. After completing the training using the FR sessions, a lever-press task in a PR schedule started. The operant requirement of each trial increased exponentially following the integer (rounded off) of 5×exp(R×0.2)−5 , where R is equal to the number of food rewards already earned plus 1 (that is, the next reinforcer), as: 1, 2, 4, 6, 9, 12, 15, 20, 25, 32, 40, 50, 62, 77, 95, and so on (Richardson and Roberts, 1996). The final ratio completed represented a breakpoint (Hodos, 1961).

### Computational models

We constructed four types of RL models (Sutton and Barto, 2018) for a lever-press task in a PR schedule. The model had three behavioral choices based on our experimental findings (Figures 1C,F): a reinforced active lever press (ALP), a non-reinforced inactive lever press (ILP), and a magazine nose poke (MNP). The four models were modifications of a standard Q-learning model (Watkins and Dayan, 1992). (1) The simple Q-learning model has single state value (hereafter, “SimpleQ”). (2) The asymmetry model has independent learning rates for positive and negative reward prediction errors (Lefebvre et al., 2017; Katahira et al., 2017b; Ohta et al., 2021). (3) The perseverance model has a choice auto-correlation to incorporate perseverance in action selection (Lau and Glimcher, 2005; Katahira, 2018). (4) The no-learning perseverance model (“NoLearn”) has a constant learning rate of zero (Katahira et al., 2017a). These models have an action value $Q_{i}^{a}$ , where the subscript i is the trial number and the superscript a is for an action $a\in \left\{\mathrm{ALP,}\;\mathrm{ILP,}\;\mathrm{MNP}\right\}$ . We assigned initial action values $Q_{0}^{a}$ as free parameters because we assumed that mice would have initial preferences among the choices in the PR task due to pretraining in FR schedules (Katahira et al., 2017a). These models updated an action value $Q_{i}^{a}$ for a chosen action according to


$$\delta_{i}=r_{i}-Q_{i}^{a},$$

$$Q_{i+1}^{a}=Q_{i}^{a}+\alpha \cdot \delta_{i}$$

where $\delta_{i}$ is the RPE, $r_{i}$ is the outcome (reward) at trial i , and α is the learning rate, which determines the weight to update action values. The outcome $r_{i}$ was binarized as 1 for a food reward and 0 for otherwise. The asymmetry model had two learning rates $\alpha_{+}$ and $\alpha_{-}$ for positive and negative RPEs, respectively. The perseverance model had additional free parameters $C_{i}^{a}$ that represent the choice trace for action a, which quantifies how frequently action a was chosen recently. The choice trace was computed according to the update rule (Akaishi et al., 2014): $C_{i+1}^{a}=C_{i}^{a}+\tau \cdot \left(1\left(a_{i}=a\right)-C_{i}^{a}\right),$ where the indicator function $1\left(a_{i}=a\right)$ assumes a value of 1 if the chosen action $\alpha_{i}$ at trial i is equal to an action a∈{ALP,ILP,MNP} . Otherwise, it takes a value of 0. The parameter τ is the decay rate of the choice trace. Initial values for the choice trace $C_{0}^{a}$ were set to zero. The NoLearn model had a constant learning rate α=0 .

The probability of choosing an action a by the models at trial i was calculated using the softmax function: $P_{i}\left(a_{i}\right)\propto \exp\left(\beta \cdot Q_{i}^{a_{i}}+\phi^{a}\cdot C_{i}^{a_{i}}\right)$ , where β is the inverse temperature parameter, and $\phi^{a}$ is the choice-trace weight for an action a that controls the tendency to repeat (when positive) or avoid (when negative) the action. Only the perseverance model and the NoLearn model had the parameters, choice trace $C_{i}^{a_{i}}$ and choice-trace weight $\phi^{a}$ .

### Parameter fitting of these models for behavioral data

Model comparisons were performed based on predictive performance of the models (Palminteri et al., 2017). Maximum log-likelihood estimation was used to fit free parameters of these models to mouse choice behavior during a PR session. The likelihood L was calculated with the formula: $L=\overset{N_{b}}{\underset{i=0}{\prod }}P_{i}\left(a_{i}\right)$ , where $N_{b}$ denotes the last trial number in a PR session, which is equivalent to the ordinal values of a breakpoint. Non-linear optimization was performed to search the most appropriate parameters using the function “optim” in the R programming language. The free parameters $Q_{i}^{a}$ and τ had lower and upper bounds from −1 to 1. The Akaike information criterion (AIC) was used to compare model fitness to the choice behavior (Daw et al., 2011): $\mathrm{AIC}=-2\log\left(L\right)+2\cdot N_{\mathrm{param}}$ , where $N_{\mathrm{param}}$ is the number of free parameters to fit in the models. Free parameters for SimpleQ, asymmetry, perseverance, and NoLearn models were 5, 6, 8, and 7, respectively. The best-fitting parameter values for each model are shown in Table 1. The model with the smallest AIC was designated as the best model (perseverance model). Please refer to Symonds and Moussalli (2011) on AIC.

**Table 1:** Fitted parameters for reinforcement learning models in lever-press tasks using a progressive ratio schedule in mice.

Model	α	$\alpha_{+}/\alpha_{-}$	*β*	$Q_{0}^{ALP}$	$Q_{0}^{MNP}$	$Q_{0}^{ILP}$	$\varphi^{ALP}$	$\varphi^{MNP}$	$\varphi^{ILP}$	AIC
SimpleQ	−11.7 ± 1.7	NA	44.7 ± 12.9	0.70 ± 0.09	0.54 ± 0.08	0.27 ± 0.08	NA	NA	NA	1,020 ± 183
Asymmetry	NA	−14.4 ± 8.2/−29.7 ± 7.5	20.2 ± 4.0	0.90 ± 0.04	0.61 ± 0.08	0.44 ± 0.07	NA	NA	NA	1,020 ± 183
Perseverance	−13.1 ± 2.6	NA	14.8 ± 2.2	0.82 ± 0.05	0.73 ± 0.05	0.23 ± 0.06	−0.1 ± 0.5	−16.6 ± 2.8	3.0 ± 0.9	*790 ± 134
NoLearn	NA	NA	28.3 ± 18.8	0.86 ± 0.01	0.79 ± 0.02	0.23 ± 0.03	−2.6 ± 2.1	−22.5 ± 6.3	2.5 ± 1.1	798 ± 134

Asterisk (*) indicates the smallest AIC for the best model.

#### Model free run

Generative performance of the best model was assessed by a free-run simulation of the three-choice behavior of mice during a PR task using the best model with its best-fitting parameter values (Palminteri et al., 2017; Wilson and Collins, 2019). The last trial number $N_{b}$ was adopted from a PR session of a mouse K11.

#### Parameter recovery and correlation

Parameter recovery simulation was performed to assess fitting of free parameters of the winning model (Wilson and Collins, 2019). Following the generation of fake choice behavior by the winning model using arbitrary chosen parameter values, we tried to recover the parameters by fitting the best model to the generated data. Association between recovered parameters and true values was checked with Pearson’s correlation coefficients.

### Fiber photometry

Extracellular DA fluctuations were measured using our custom-made fiber photometric system (Natsubori et al., 2017; Shikano et al., 2023). Extracellular DA fluorescence signals were obtained by illuminating cells that expressed GRAB_DA2m_ with a 465 nm LED (8.0 ± 0.1 μW at the patch cable tip) and a 405 nm LED (8.0 ± 0.1 μW at the patch cable tip). The 465 nm and 405 nm LED lights were emitted alternately at 20 Hz (turned on for 24 ms and off for 26 ms), with the timing precisely controlled by a programmable pulse generator (Master-8, A.M.P.I., Jerusalem, ISRAEL). Each excitation light was reflected by a dichroic mirror (DM455CFP; Olympus) and coupled into an optical fiber patch cable (400 μm in diameter, 2 m in length, 0.39 NA, M79L01; Thorlabs, Newton, NJ) through a pinhole (400 μm in diameter). The optical fiber patch cable was connected to the optical fiber cannula of the mice. The fluorescence signal was detected by a photomultiplier tube with a GaAsP photocathode (H10722–210; Hamamatsu Photonics, Shizuoka, Japan) at a wavelength of 525 nm. The fluorescence signal, TTL signals that specified the duration of the 465 or 405 nm LED excitations, and TTL signals from behavioral settings were digitized by a data acquisition module (cDAQ-9178, National Instruments, Austin, TX) with a voltage input module (NI-9215, National Instruments). The group of digitized signals was simultaneously recorded at a sampling frequency of 1,000 Hz by a custom-made program (LabVIEW 2016, National Instruments). The fluorescence signal was processed offline, yielding a ratiometric 465/405 signal at a frame rate of 20 Hz, which represented extracellular DA concentration (Shikano et al., 2023). The processed ratiometric signal trace was high-pass filtered at approximately 0.0167 Hz, corresponding to a wavelength of 1 min to exclude low-frequency fluctuations. We calculated mean and standard deviation of the DA signal using the last 20 s (66%) of the ITI period prior to a trial start of every trial in a session to obtain *z*-scores of the DA signal. Using only the latter part of ITI was important for the calculation of *z*-scores because DA fluctuation during the first half of ITI may be contaminated by DA fluctuations induced by food consumption. Although we chose the latter part of ITI as the DA baseline because DA levels seemed relatively stable during lever-press tasks, it is still possible that mice may have anticipated lever extensions toward the end of ITI, accompanying a slight DA increase. To generate peri-event plots for a magazine nosepoke and reward, DA fluctuations were binned temporally into blocks of 100 ms. Amplitudes of a DA dip trough upon magazine nosepokes and a DA surge upon food rewards were obtained as minimum or maximum of DA signals (*z*-scores) during 3 s periods after each event.

### Histology

After the completion of the behavioral task, the location of an optical fiber insertion and expression pattern of GRAB_DA2m_ protein in the striatum was assessed with a brain slice (Supplementary Figure S3). Mice were subjected to the same anesthesia described in the surgery section and were intracardially perfused with 4% paraformaldehyde phosphate buffer solution. Brains were removed and cryoprotected in 20% sucrose overnight, frozen, and cut into 50 μm thick sections on a cryostat (Leica CM3050 S, Leica Biosystems, Wetzlar, Germany). Sections were mounted on silane-coated glass slides (S9226, Matsunami Glass, Osaka, Japan). The GRAB_DA2m_ signals received no amplification. Fluorescence images were captured with an all-in-one microscope (BZ-X710, Keyence, Osaka, Japan).

## Results

### Void magazine nosepoke is a major behavioral choice in a progressive ratio lever-press task

Lever-press tasks for mice have been commonly treated as two-choice tasks with active and inactive lever presses (Ito and Doya, 2015; Tsutsui-Kimura et al., 2017b), but recent studies suggested a void magazine nosepoke, which is a checking behavior by mice of a food magazine before completing the required number of lever presses in a PR schedule of reinforcement, as a third behavioral choice during lever-press tasks (Wanat et al., 2013; Ko and Wanat, 2016; Zhou et al., 2022). Indeed, we incorporated an magazine nosepoke as the third choice in our RL model for a lever-press task in an FR5 schedule, demonstrating the relation between an RPE in the model and the frequency of a magazine nosepoke by mice (Shikano et al., 2023). Therefore, we asked whether a magazine nosepoke is also a major behavioral choice in a lever-press task in our PR schedule.

Five mice were trained first to associate active lever pressing and a food pellet reward with lever-press tasks in FR1 and FR5 schedules. After establishing the association, mice performed a lever-press task in a PR schedule once per day for 6–8 days (Figure 1A). We observed that mice frequently chose an magazine nosepoke in addition to active and inactive lever presses during the PR session (Figure 1B). Mice received a reward pellet from the magazine when a sufficient number of active lever presses for a trial were achieved (vertical orange lines for reward in Figure 1B). Magazine nosepokes occurred intermittently, rather than continuously, with other choice behaviors interspersed (Figure 1C). The maximum number of active lever presses for one reward in a session, which is defined as a breakpoint, was from 20 to 402, resulting in 8 to 22 pellet rewards in a single session (Figure 1D). In the modeling analysis in the following sections, behavioral data for PR tasks from days 3 to 8 were used for stable performance of PR tasks (Figure 1E). Fluctuation of a breakpoint during days 3 to 8 was smaller than 15% without a significant difference (one-way repeated-measures ANOVA, *F*_[4,20]_ = 0.55, *p* = 0.698). Total counts of magazine nosepokes (183 ± 41, *n* = 10 mice, 30 sessions) were comparable to those of other behavioral choices (reward, 15.2 ± 1.3; active lever press, 885 ± 224; inactive lever press, 41 ± 16) (Figure 1F; Supplementary Figure S1), suggesting that it is important to incorporate magazine nosepokes as one of the behavioral choices in a reinforcement learning model for a lever-press task in a PR schedule for mice.

### Reinforcement learning model with a perseverance factor best replicated a choice behavior during a PR session

**Figure 2:**
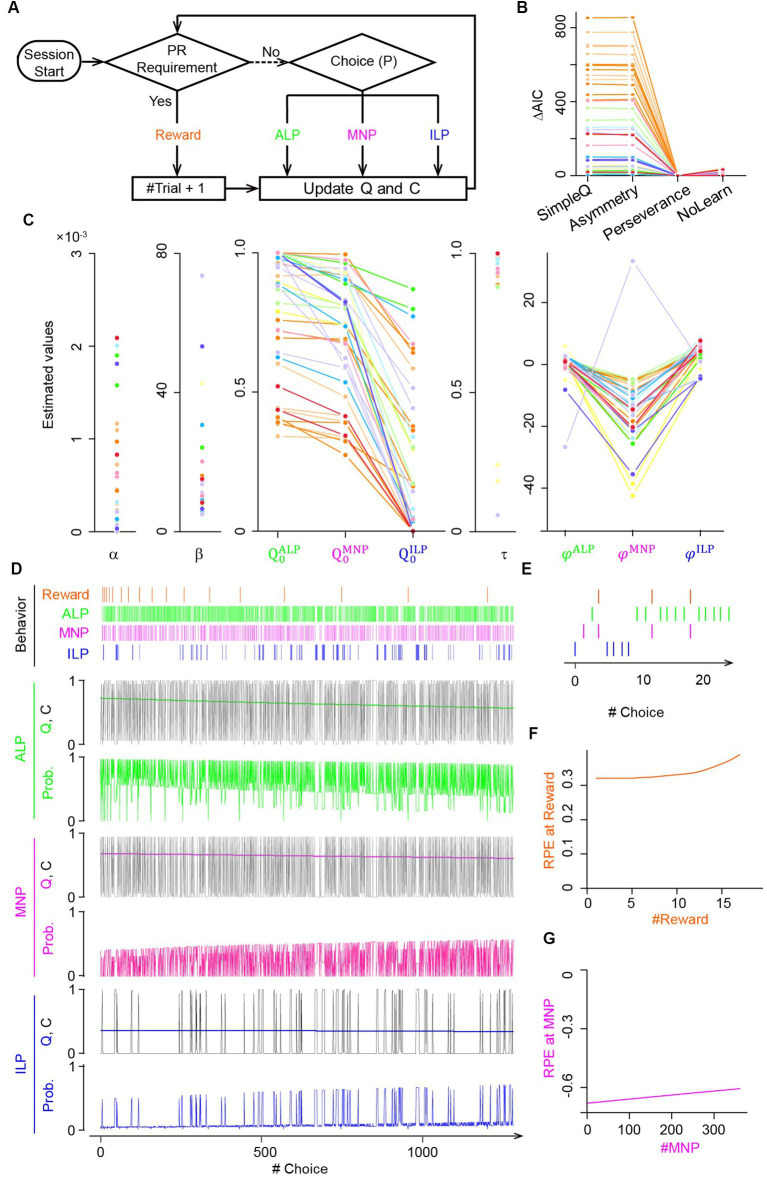
Q-learning model with choice traces predicted modulation of reward prediction errors during the progressive ratio lever-press task. **(A)** PR lever-press task for reinforcement learning models. An agent of the Q-learning models chooses an active lever press (ALP), a magazine nosepoke (MNP), or an inactive lever press (ILP), followed by updates of action values *Q* and/or a choice trace *C* until the PR requirement is met. **(B)** Comparison of goodness-of-fit of four Q-learning models for the PR lever-press task: (1) the SimpleQ model with a learning rate *α*, (2) the asymmetry model with two learning rates, *α*_+_ and *α*_−_, for positive and negative RPEs, respectively, (3) the perseverance model with a learning rate *α* and choice-trace weights $\varphi^{\mathrm{ALP}}$ , $\varphi^{\mathrm{MNP}}$ , and $\varphi^{\mathrm{ILP}}$ for choice traces *C*^ALP^, *C*^MNP^, and *C*^ILP^, respectively, for modeling the effects of choice hysteresis, and (4) the NoLearn model, which is the perseverance model, with a constant learning rate *α* = 0. AIC was calculated for the four Q-learning models fitted to mouse behavioral data. Lines of the same hue represent data from the same mice. The *y*-axis represents differences of AIC from the perseverance model. The AIC value of the perseverance model was significantly smaller than that of other models, indicating the perseverance model as the best model (paired *t*-test comparing AIC values of the perseverance model and comparable models: *p* = 0.003, 0.003, and 0.002 for SimpleQ, asymmetry, and NoLearn models, respectively). Lines connect the same session of a mouse (38 sessions from 13 mice). **(C)** Values of free parameters in the perseverance model, fitted to mouse behavior. The initial action value for a magazine nosepoke (MNP), $\varphi_{0}^{\mathrm{MNP}}$ , was comparable to that for an active lever press, leading to frequent magazine nosepokes by mice during PR sessions. Choice-trace weights for a magazine nosepoke, $\varphi^{\mathrm{MNP}}$ , were significantly smaller than for active and inactive lever presses, suggesting a lower tendency for consecutive magazine nosepokes. **(D)** Free-run simulation of the perseverance model. A representative choice pattern (top row) resembles that of actual mice (Figure 1B). Subsequent rows show time series of action values *Q*, choice traces *C*, and choice probabilities for each action active lever press (ALP, green), magazine nosepoke (MNP, magenta), and inactive lever press (ILP, blue) during the simulation of PR sessions. Action values *Q* for active lever press (green) and magazine nosepokes (magenta) were comparable, reflecting association of lever-pressing and a reward delivery. Choice trace *C* modulates the probability (Prob.) of choosing an action by increasing the input values to the softmax function. Probabilities for actions are complementary and sum to 1. **(E)** Expanded view of the free-run simulation for the first 20 choices by the perseverance model. **(F,G)** The perseverance model predicted increasing **(F)** and decreasing **(G)** amplitudes in RPEs upon a reward delivery (reward, orange) and a magazine nosepoke (MNP, magenta), respectively, over the course of the PR session.

We constructed four RL models with three behavioral choices (active and inactive lever presses, and magazine nosepokes) to assess choice behavior during a PR lever-press task. The agent of the RL models followed the steps in Figure 2A: an agent checked first whether a PR requirement was fulfilled, or a sufficient number of active lever presses was performed. If the PR requirement was fulfilled, an agent received a reward and the trial number was incremented, followed by updates of a state value $Q_{i}^{\mathrm{MNP}}$ and a choice trace $C_{i}^{\mathrm{MNP}}$ if present. When the requirement was not fulfilled, an agent chose a behavior among an active lever press, a magazine nosepoke, and an inactive lever press, followed by updates of the state value $Q_{i}$ and the choice trace $C_{i}$ for a chosen behavior reflecting its outcome (no reward). An agent repeated these steps for actual times in a PR session by mice.

The four RL models were (1) SimpleQ, (2) asymmetry, (3) perseverance, and (4) NoLearn models (Figure 2B). The SimpleQ model is most commonly used in model-based analysis of choice behavior (Katahira et al., 2017b). The asymmetry model had distinct learning rates for positive and negative RPEs, respectively. The reason to investigate the asymmetry model is that repeated behavioral choices required for an active lever press to obtain a reward in the current PR tasks may resemble scarce environments (Ohta et al., 2021). A recent study demonstrated that rodents in scarce-reward environments had uneven learning rates with a ratio $\alpha_{+}/\alpha_{-}$ of about 10, indicating that an agent updates values 10 times more with a positive RPE, i.e., obtaining a reward, than a negative RPE, i.e., no reward (Ohta et al., 2021). Fitting the asymmetry model to actual mouse behavior during PR tasks resulted in a learning rate ratio $\alpha_{+}/\alpha_{-}$ of 1.6 ± 1.2 (fitted to behavioral data from 13 mice with 38 sessions; $\alpha_{+}$ , −14.4 ± 8.2; $\alpha_{-}$ , −29.7 ± 7.5), which was significantly smaller than the ratio for scarce-reward environments, implying that mice did not regard PR tasks as scarce environments. AIC values of the asymmetry model were comparable to those of the SimpleQ model, suggesting that introduction of asymmetric learning rates did not increase fitting of the choice behavior of PR lever-press tasks (Figure 2B). The third model, the perseverance model, incorporated a choice trace to represent perseverance in action selection (Lau and Glimcher, 2005; Akaishi et al., 2014). The perseverance model was investigated because a simulation study demonstrated that a model without a choice trace could wrongly assign asymmetric learning rates for perseverance behavior (Katahira, 2018; Sugawara and Katahira, 2021) and because we observed a persistent behavior of mice during PR tasks (Figure 1B). The AIC of the perseverance model was significantly lower than that of the SimpleQ and asymmetry models (Figure 2B), implying that repeated active lever presses in PR tasks are better described as persistence rather than asymmetric learning (difference of AIC values of a model from that of the perseverance model. *p*-values obtained by a paired *t*-test: SimpleQ, 1,016 ± 183, *p* = 0.003; asymmetry, 1,015 ± 183, 0.003; *n* = 13 mice, *n* = 38 sessions) (Lau and Glimcher, 2005; Schönberg et al., 2007; Katahira, 2015). The perseverance model showed stable action values *Q* during PR sessions (Figure 2D, *Q* values for an active lever press, a magazine nosepoke, and an inactive lever press), reflecting its small learning rate (Figure 2C α ). This small learning rate implied that a perseverance model with a constant learning rate α=0 (NoLearn model) might be enough for PR tasks. Therefore, we compared AICs for perseverance and NoLearn models (Figure 2B), obtaining a significantly smaller AIC for the perseverance model (Figure 2B. Difference of AICs between NoLearn and perseverance models, 8.4 ± 2.2, *p* = 0.002). Thus, a small, positive learning rate was necessary to describe PR tasks. In conclusion, the perseverance model achieved the best predictive performance in the PR lever-press tasks for mice (Figure 2B).

Fitted parameters of the perseverance model to behavioral data of mice are shown in Figure 2C and summarized in Table 1 with other models, demonstrating a consistent tendency in parameter fitting (Figure 2C. *n* = 13 mice; learning rate α (6.8 ± 2.1) × 10^−4^; inverse temperature β 14.3 ± 2.3; initial state values $Q_{0}^{\mathrm{ALP}}$ 0.91 ± 0.04, $Q_{0}^{\mathrm{MNP}}$ 0.77 ± 0.04, $Q_{0}^{\mathrm{ILP}}$ 0.69 ± 0.04; decay rate of the choice-trace weight τ 0.69 ± 0.04; choice-trace weight $\varphi^{\mathrm{ALP}}$ −0.39 ± 0.80, $\varphi^{\mathrm{MNP}}$ −13.9 ± 2.0, $\varphi^{\mathrm{ILP}}$ 3.1 ± 0.6). Specifically, the initial state value for a magazine nosepoke was significantly larger than that for ILP, further supporting the notion that a magazine nosepoke constitutes a major behavioral choice during lever-press PR sessions for mice (*n* = 13 mice, *t*-test, *p* = 2.6 × 10^−6^). Choice-trace weights φ for a magazine nosepoke were negative (except for a mouse indicated in a gray line in Figure 2C) and significantly smaller than that for active or inactive lever presses, implying that mice had a tendency to avoid consecutive magazine nosepokes.

Generative performance of the perseverance model was checked by performing a free run of the model with the best-fitting parameter values (Palminteri et al., 2017). Time series of simulated choice behavior of the perseverance model (Figure 2D) were similar overall to actual mouse behavior during PR tasks (Figure 1B). The perseverance model succeeded in replicating characteristic mouse choice behavior (Figure 1D): repetitive active lever presses and continual magazine nosepokes with intervals (Figure 2E). We found gradual increases and decreases of RPEs upon reward delivery (Figure 2F) and magazine nosepokes without reward deliveries (Figure 2G), reflecting gradual decreases of action values for magazine nosepokes during PR sessions ( $Q_{i}^{\mathrm{MNP}}$ in Figure 2D). These results were used to make predictions about corresponding DA dynamics in the striatum of actual mice because of the proposed relation between RPEs and DA dynamics (Schultz et al., 1997).

We checked the validity of parameter fitting of the perseverance model with parameter recovery experiments (Supplementary Figure S2) (Wilson and Collins, 2019). We used Pearson’s correlation coefficients to confirm that it was able to recover pre-set parameter values by fitting the perseverance model to the choice behavior sequence obtained by free running the perseverance model with pre-set parameter values. Correlation coefficients were comparable to previous reports (Supplementary Figure S2A: 220 simulations; correlation coefficients for parameters: α , 0.502; β , 0.383; τ , 0.777; $Q_{0}^{\mathrm{ALP}}$ , 0.450, $Q_{0}^{\mathrm{MNP}}$ , 0.433; $Q_{0}^{\mathrm{ILP}}$ , 0.583; $\varphi^{\mathrm{ALP}}$ , 0.670; $\varphi^{\mathrm{MNP}}$ , 0.545; $\varphi^{\mathrm{ILP}}$ , 0.654), suggesting satisfactory parameter recovery (Daw et al., 2011). We also confirmed that there was no significant correlation among recovered parameters (Supplementary Figure S2B), suggesting that free parameters in the perseverance model were independent. These results support the feasibility of parameter fitting and model construction.

### Predictions of the perseverance model on reward prediction errors during the PR task were corroborated by dopamine dynamics in the ventral striatum of mice

Our perseverance model predicted gradual increases and decreases of RPE amplitudes upon a reward delivery and a magazine nose poke, respectively, over the course of PR task execution. Because DA is suggested to be a neuronal implementation of RPEs in the brain (Schultz et al., 1997; but see Hnasko et al., 2005 for learning without DA), we measured extracellular DA in the VS of mice during PR lever-press tasks to validate predictions of the perseverance model.

**Figure 3:**
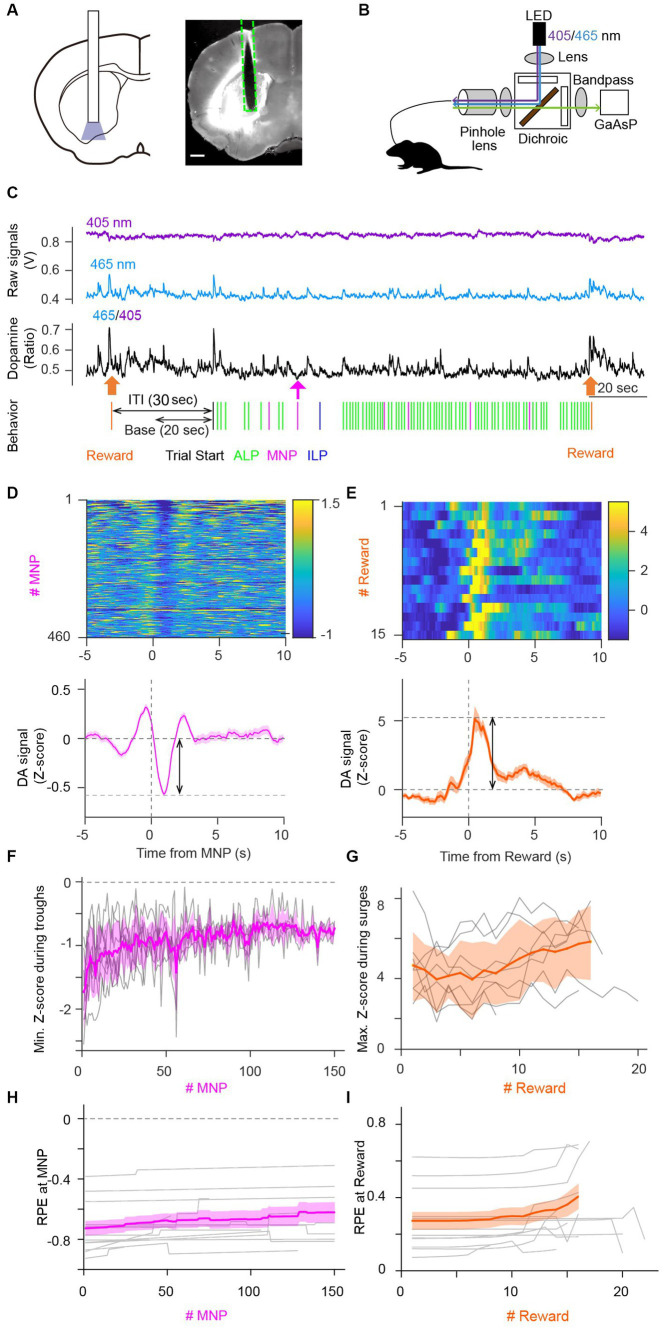
Dopamine dynamics in the ventral striatum during the PR task validated predictions of the Perseverance model. **(A)** Schematic illustration of optical fiber insertion into the VS of mice (left) and a representative mouse brain coronal section showing the expression pattern of a fluorescent DA sensor, GRAB_DA2m_, (white area) with the optical fiber track (dashed line, right). Scale bar, 500 μm. **(B)** Fiber photometry system. A single multimode fiber was connected to the optical fiber implanted in the VS. Excitation light was applied continuously with alternating wavelengths of 405 nm (purple) and 465 nm (blue). Fluorescence emissions at 525 nm (green) were detected. **(C)** Representative GRAB_DA2m_ signal dynamics during a PR lever-press task. Fluorescence intensity fluctuations of GRAB_DA2m_ excited by 405 nm (purple) or 465 nm (blue) light were divided to obtain a ratio (465/405, black) as a proxy for extracellular DA concentration. Accompanying choice behavior of the mouse during the PR task is shown at the bottom. Reward delivery (orange arrow) induced a large DA surge. An magazine nosepoke (MNP, magenta arrow) accompanied a small transient DA decrease. The latter part of inter-trial interval (ITI) was used as the base period to normalize the DA signal. **(D,E)** Representative heatmaps showing DA signal fluctuations aligned to magazine nosepokes (MNP) **(D)** or rewards **(E)** in a session (top). Averaged DA time courses for heatmaps demonstrate a transient DA decrease at a magazine nosepoke (**D**, bottom), which coincided with the negative RPE upon an magazine nosepoke in the perseverance model and a DA surge at reward (**E**, Bottom), which coincided with the positive RPE upon a reward delivery (**E**, bottom). Double arrows indicate amplitudes of transient DA dynamics. **(F,G)** Amplitudes of the transient DA decrease upon magazine nosepokes and the transient DA surge upon reward became smaller **(F)** and larger **(G)** during a PR session, replicating the prediction of free-run simulation of the perseverance model (Figures 2F,G). Gray lines, DA dynamics of each session of mice. Thick lines in magenta or orange, mean DA dynamics. A transparent area in magenta or orange indicates the standard deviation of DA signal. **(H,I)** Time courses of RPEs upon magazine nosepokes **(H)** or rewards **(I)** of the perseverance model that were fitted to choice behavior of each session of mice during PR tasks. Slight decreases **(H)** and increases **(I)** of RPEs upon a magazine nosepoke and reward, respectively, were consistent with the free-run simulation of the perseverance model (Figures 2F,G) and with DA dynamics of mice during PR tasks **(F,G)**. Gray lines, RPE dynamics of the perseverance model fitted to mouse choice behavior of a session. Thick lines in magenta or orange mean RPEs upon magazine nosepokes or rewards. A transparent area in magenta or orange indicates the standard deviation of RPEs.

We injected an AAV virus to express a genetically encoded optical DA sensor—GRAB_DA2m_—in the VS (Figure 3A). The VS is involved in lever-pressing operant tasks under FR schedules in terms of intracellular calcium activity (Natsubori et al., 2017; Tsutsui-Kimura et al., 2017a,b; Yoshida et al., 2020) and extracellular DA dynamics (Shikano et al., 2023). Our custom optical fiber system enabled monitoring of extracellular DA level fluctuations in the VS during PR sessions (Figure 3B) (Shikano et al., 2023). Ratiometric calculation of GRAB_DA2m_ fluorescence signals excited at 405 nm and 465 nm corresponded to extracellular DA dynamics (Figure 3C). Ratio metric calculation helped to distinguish DA decreases and artifactual fluorescence drops due to fluorescence bleaching or body movements. There were large DA increases upon reward delivery and small DA decreases upon magazine nosepokes (Figure 3C), consistent with previous studies (Ko and Wanat, 2016; Shikano et al., 2023). A representative heatmap demonstrated a small, but clear decrease and a large increase in DA fluctuation upon a magazine nosepoke (negative peak value −0.57 ± 0.57 in mean ± standard deviation, *n* = 460 trials, two-tailed *t*-test, *p* < 0.0001, Figure 3D) and reward delivery (positive peak value 5.2 ± 3.4 in mean ± standard deviation, *n* = 15 trials, two-tailed *t*-test, *p* < 0.0001, Figure 3E), respectively. Transient decreases of DA 1–2 s after magazine nosepokes are consistent with previous reports (Figure 3D lower panel) (Ko and Wanat, 2016). Amplitudes of a DA surge upon unconditioned stimulus (reward delivery) were significantly larger than that of DA decrease upon magazine nosepokes, which are also consistent with previous studies (Figure 3E lower panel) (Ko and Wanat, 2016; Shikano et al., 2023). Note that a DA increase just before a magazine nosepoke may reflect reward expectation (Figure 3D) (Shikano et al., 2023).

To examine the model prediction, we plotted time series of DA dip amplitudes upon magazine nosepokes (Figure 3F). We performed linear regression to quantify the decreasing trend in DA dip amplitudes, confirming that DA dip amplitudes upon magazine nosepokes significantly decreased over the PR session (*n* = 8 mice. Count of magazine nosepokes, 216 ± 59. Linearly regressed DA dip amplitudes decreased upon an magazine nosepoke without a reward: slope, (5.4 ± 1.8) × 10^−3^; intercept, −1.1 ± 0.08; *t*-test on a slope, *p* = 0.020). We also observed increasing trends of a DA surge upon reward delivery, although the increasing trend was not significant (Figure 3G; *n* = 8 mice, session number = 24, count of a reward supply, 15.1 ± 1.2. Linearly regressed DA surge amplitudes increased upon reward delivery: slope, (5.2 ± 5.5) × 10^−2^; intercept, 4.1 ± 0.5; *t*-test on a slope, *p* = 0.374). Fitting of the perseverance model to behavioral data of mice demonstrated a decrease of DA dip amplitudes upon a magazine nosepoke (Figure 3H) and an increasing trend of DA surge amplitudes upon reward (Figure 3I). These results support the perseverance model for describing choice behavior during PR lever-press tasks, relating RPEs and DA dynamics in the brain.

### The perseverance model captured the effects of low-dose methamphetamine on choice behavior during a PR task as an increase in initial action values

Next, we asked whether the perseverance model can describe modulation of choice behavior during PR tasks by psychiatric drugs. While moderate-dose METH (1.0 mg/kg) injection before lever-press PR tasks increased the breakpoint (Thompson, 1972; Bailey et al., 2015), low-dose METH injection has not been reported to change the breakpoint (Asami and Kuribara, 1989; Hall et al., 2008; Miller et al., 2013; Jing et al., 2014). Therefore, behavioral effects of the low-dose drug are not revealed by a breakpoint in PR tasks. To advance computational understanding of modulatory effects of low-dose METH on choice behavior, we applied our perseverance model to analyze choice behavior of mice during PR tasks.

**Figure 4:**
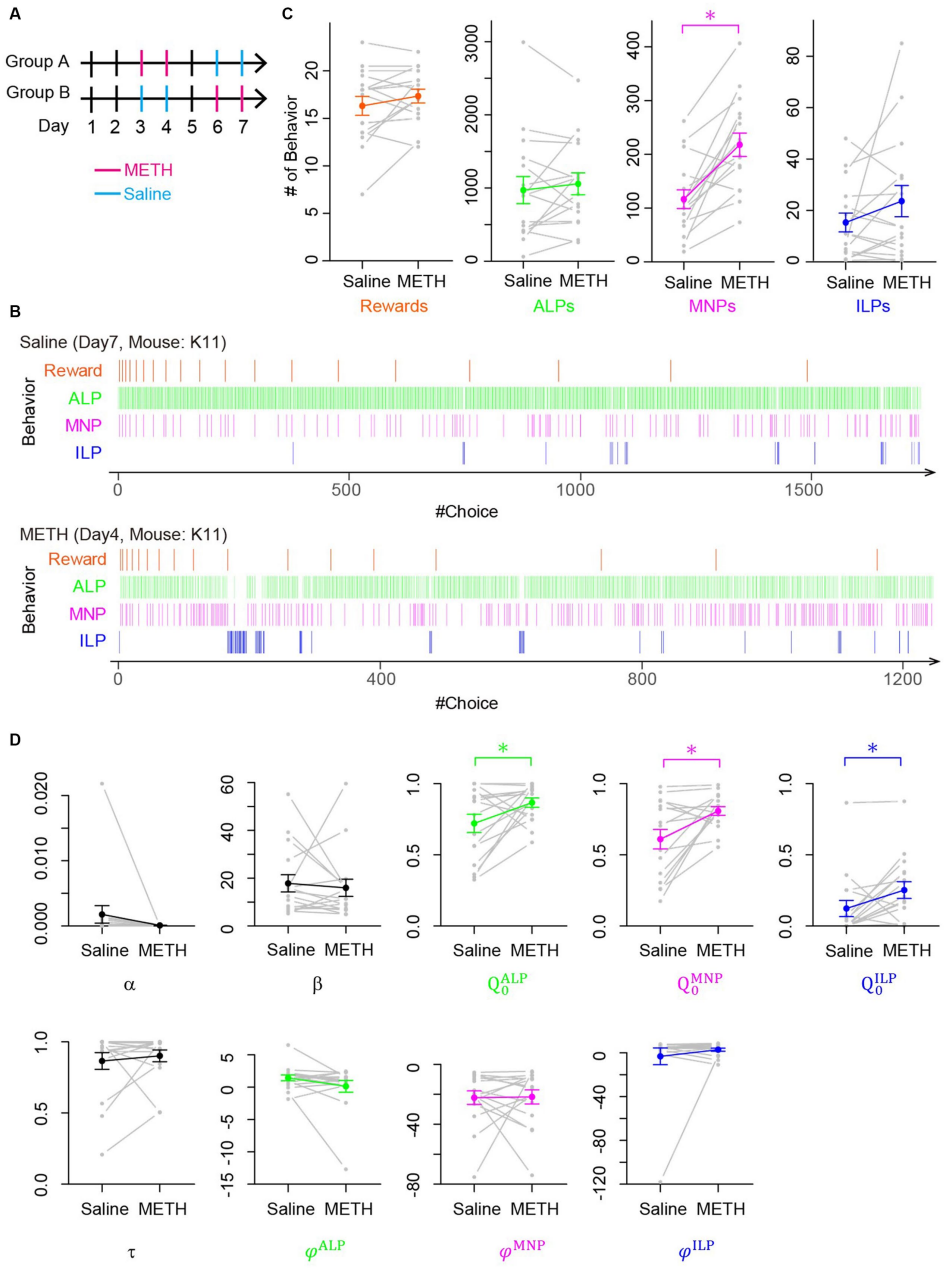
Low-dose METH modified choice behavior during a PR task without changing the breakpoint, which the perseverance model explained as increases of initial action values. **(A)** Schedule of behavioral experiments for a PR lever-press task with methamphetamine (METH, magenta) or saline (blue) injection. Mice in Group A received low-dose METH injections (0.5 mg/kg) on days 3 and 4 and saline injections on days 6 and 7. Mice in Group B received injections in an opposite manner. On days 1, 2, and 5, mice performed a PR task without injection. **(B)** Representative time course of choice behavior of mice during a PR lever-press task with an injection of saline (upper) or METH (lower). Slight increase of a magazine nosepoke frequency is visible in a mouse with low-dose METH injection. **(C)** Comparison of mouse behavior during a PR session with and without METH injection. Numbers of magazine nosepokes (MNPs) increased significantly (*n* = 16 mice, *t*-test, *p* = 0.00012). Other choices including rewards, which correspond to a breakpoint, were not modified by METH injection (*t*-test, rewards, *p* = 0.12; ALP, *p* = 0.44; ILP, *p* = 0.16). **(D)** Comparison of free parameters of the perseverance model that was fitted to choice behavior of mice in a PR session with and without METH injection. Low-dose METH significantly increased initial action values for active lever press (ALP), MNP, and inactive lever press (ILP) (*n* = 16 mice, paired *t*-test; $Q_{0}^{\mathrm{ALP}}$ , *p* = 0.043; $Q_{0}^{\mathrm{MNP}}$ , *p* = 0.009; $Q_{0}^{\mathrm{ILP}}$ , *p* = 0.031).

We first performed behavioral experiments using mice for a lever-press PR task. After completing pretraining to associate lever presses with rewards, eight mice each were assigned to Groups A and B for PR tasks for consecutive 7 days (Figure 4A). Low-dose METH (0.5 mg/kg i.p.) was injected 10 min prior to a PR session on days 3 and 4 (Group A) or days 6 and 7 (Group B). As control experiments, saline was injected on days 6 and 7 (Group A) or days 4 and 5 (Group B). Figure 4B shows representative choice behavior during a lever-press PR session after saline (upper panel of Figure 4B) or low-dose METH (lower panel of Figure 4B) injections, demonstrating that low-dose METH increased the frequency of magazine nosepokes during a PR session (Figure 4B). We quantified choice behavior during PR tasks after METH or saline injections (Supplementary Figure S5), finding that low-dose METH injection significantly increased the number of magazine nosepokes in a session (MNPs in Figure 4C, *n* = 16 mice, *t*-test, *p* = 0.00012, 117 ± 17 for saline vs. 218 ± 22 for METH) while low-dose METH did not modify the breakpoint (number of rewards in a session in Figure 4C. *N* = 16 mice, session number = 61, *t*-test, *p* = 0.12, 16.3 ± 1.0 for saline, 17.3 ± 0.7 for METH), number of active nor inactive lever presses (Figure 4C. *N* = 16 mice, *t*-test; active lever press: *p* = 0.44, count of active lever presses, 971 ± 185 for saline and 1,057 ± 150 for METH; ILP: *p* = 0.16, count of inactive lever presses, 15.3 ± 3.7 for saline and 23.6 ± 6.1 for METH).

We fitted our perseverance model to the choice behavior of mice during PR tasks after saline or low-dose METH injection to investigate computational modulation by low-dose METH. We found that low-dose METH increased initial action values for an active lever press, a magazine nosepoke, and an inactive lever press significantly (Figure 4D; *n* = 16 mice, paired *t*-test; $Q_{0}^{\mathrm{ALP}}$ , *p* = 0.043, 0.721 ± 0.064 vs. 0.866 ± 0.032, $Q_{0}^{\mathrm{MNP}}$ , *p* = 0.009, 0.610 ± 0.068 vs. 0.808 ± 0.030, $Q_{0}^{\mathrm{ILP}}$ , *p* = 0.031, 0.123 ± 0.057 vs. 0.252 ± 0.059 for saline vs. METH, respectively), while other free parameters were not modulated (*n* = 16 mice, *t*-test: α , *p* = 0.88, −19.2 ± 9.0 vs. −20.9 ± 5.0, β , *p* = 0.68, 17.8 ± 3.6 vs. 15.9 ± 3.6; *τ*, *p* = 0.52, 0.865 ± 0.059 vs. 0.902 ± 0.041; *φ*^ALP^, *p* = 0.14, 1.43 ± 0.45 vs. 0.12 ± 0.90; *φ*^MNP^, *p* = 0.94, −22.3 ± 4.5 vs. −21.8 ± 4.7; *φ*^ILP^, *p* = 0.42, −3.2 ± 7.7 vs. 2.8 ± 1.4). These results imply that low-dose METH increased frequency of magazine nosepokes during PR sessions by augmenting initial action values, rather than by learning-related free parameters such as *α* and *β*.

## Discussion

In this study, we proposed an RL model to analyze choice behavior during a lever-press PR task in male mice. We demonstrated that choice traces are critical to incorporate perseverance in action selection during PR tasks, rather than asymmetric learning rates. While PR tasks have been widely used to quantify motivation with a breakpoint, this method does not allow assessment of choice behavior during PR sessions because breakpoints are calculated after completing the PR session as the largest number of active lever presses achieved for a reward during the session. Our perseverance model is unique in having a behavioral choice for magazine nosepokes in addition to conventional choices for active and inactive lever presses. Incorporation of magazine nosepokes into an RL model was critical in this study because low-dose METH modulated the frequency of magazine nosepokes without changing the breakpoint. The perseverance model predicted a gradual decrease of RPE amplitudes upon a magazine nosepoke without a reward delivery during the PR session. We validated the prediction experimentally using fluorescence measurements of extracellular DA in the VS during PR tasks for mice, relating the perseverance model and neurophysiology. We showed application of the perseverance model on low-dose METH. The perseverance model demonstrated that the increase of magazine nosepokes during a PR session by METH injection was caused by increased initial action values. The perseverance model would be a useful tool to investigate the effects of psychoactive drugs on choice behavior during lever-press PR tasks.

Initial action values were set as free parameters in our model, while they are frequently set to zero in RL models (Katahira, 2015). The rationale for setting initial action values to zeros is that agents of RL models choose actions and update action values repeatedly, which decreases the contribution of initial action values to zero asymptotically. In addition, RL models with fewer free parameters are generally preferable (Wilson and Collins, 2019). In the present study, however, initial action values were indispensable to capture the effect of low-dose METH on a choice behavior during PR tasks. The reason for the significance of initial action values in the present study would be the small learning rate. Due to small learning rates, action values did not change dramatically by value updates upon a choice behavior during a PR session. It is noteworthy that small, but positive learning rates were still necessary for describing choice behavior. The small, but positive learning rate in the perseverance model allows the model to adapt to a situation in a PR task in which the requirement for lever-press counts increases exponentially during a session. Therefore, we presented a unique situation in an RL model for mice, in which modulation of initial action values, rather than learning-related parameters such as a learning rate, resulted in bias in choice behavior (Biele et al., 2011). It is intriguing that not only the initial action value for a magazine nosepoke but also that for active and inactive lever presses were increased by low-dose METH. These results may coincide with previous reports that acute amphetamine disrupted discrimination of cues with different reward sizes (Werlen et al., 2020). The neurophysiological substrate for increased initial action values is not clear in this study. Increases of tonic DA concentration by METH injection might be related to initial action value modulation because we have shown that moderate- and high-dose METH injection increases extracellular DA concentration in the mouse VS over an hour (Iino et al., 2020). Functional magnetic resonance imaging (fMRI) might be fruitful to reveal brain regions affected by low-dose METH injection (Taheri et al., 2016; Yoshida et al., 2016; Weafer et al., 2019). An fMRI study on human subjects suggested encoding of RPE at the striatum and action values at the medial prefrontal cortex, respectively (Bernacer et al., 2013).

One limitation of the perseverance model is that it does not describe a breakpoint in PR tasks. RL models including our perseverance model are suited for describing choice behavior but commonly do not have a mechanism to stop choosing a behavior. A previous study incorporated a motivation factor in an Actor-Critic RL model for describing thirstiness of the agent in a visual GO/NOGO lick task for mice, succeeded in describing a response rate decrease in the late phase of a session (Berditchevskaia et al., 2016). The motivation factor was an additional positive value to bias an action value for GO choice, which diminishes every time a water reward is received. In that study, a decrease of a GO choice and an increase of a NOGO choice during the late phase of a session were described successfully by the motivation factor. However, in the current PR tasks, mice stop making behavioral choices at a breakpoint. Therefore, simple introduction of the motivation factor into the current perseverance model cannot identify breakpoints. Mathematical Principles of Reinforcement (MPR) models have been proposed to describe relationships between response requirements and subsequent behavioral pauses, enabling prediction of breakpoints based on regressed parameters (Killeen et al., 2009; Bradshaw and Killeen, 2012). The MPR model is, however, a descriptive model that does not provide a computational basis for each choice behavior in PR tasks. Another caveat is dose-dependent effects of METH. High-dose METH (3.0–10.0 mg/kg) induces stereotyped behavior in mice such as focused sniffing, licking, or grooming (Kelley, 2001; Mason and Rushen, 2008; Shen et al., 2010) not performing choice behavior for PR tasks (Randrup and Munkvad, 1967; Hadamitzky et al., 2012). The perseverance model cannot be applied to such situations. Notably, the behavioral test was performed using only the right side of the ventral striatum of male mice; thus, generalizability of the current results still requires verification.

In conclusion, we highlighted the importance of magazine nosepokes as a behavioral choice during PR tasks that indicates mouse expectation of a reward. Our perseverance model analyzes choice behavior during a PR task that expands utility of PR tasks and advances understanding of computational mechanisms of effects by psychoactive drugs that cannot be revealed with a breakpoint.

## Data availability statement

The raw data supporting the conclusions of this article will be made available by the authors, without undue reservation.

## Ethics statement

The animal study was approved by the Animal Ethics Committee of Keio University. The study was conducted in accordance with the local legislation and institutional requirements.

## Author contributions

KI: Conceptualization, Formal analysis, Investigation, Validation, Visualization, Writing – original draft, Data curation. YS: Investigation, Writing – review & editing. SK: Investigation, Writing – review & editing. SY: Funding acquisition, Resources, Writing – review & editing. KT: Conceptualization, Funding acquisition, Writing – review & editing. NT: Conceptualization, Data curation, Formal analysis, Funding acquisition, Methodology, Project administration, Software, Supervision, Validation, Visualization, Writing – original draft, Writing – review & editing.
