# Supplementary material for: A reinforcement learning model with choice traces for a progressive ratio schedule
Source: Front Behav Neurosci. 2024 Jan 10;17:1302842. doi: 10.3389/fnbeh.2023.1302842 (PMC10806202; doi:10.3389/fnbeh.2023.1302842)
Supplement: Supplementary file 1 [file Data_Sheet_1.docx]

Legends for Supplementary Figures 1-5
in “A Reinforcement Learning Model with Choice Traces for a Progressive Ratio Schedule”
by Keiko Ihara, Yu Shikano, Sae Kato, Sho Yagishita, Kenji F. Tanaka, Norio Takata

# FIGURE LEGENDS

## Supplementary Figure 1. Correlation matrix of choice behaviors during PR tasks.

Pearson’s correlation coefficients between counts of reward (RW), active lever press (ALP), magazine nosepokes (MNP), and inactive lever presses (ILP) during a progressive ratio (PR) lever press task are shown in the upper triangular matrix. Each dot in the lower triangular matrix represents the mean count of a behavior per session for each mouse. Strong positive correlation was observed between RW and ALP, and MNP and ILP, respectively. The image was generated using the chart.Correlation function from the PerformanceAnalytics package in R. Significance of the correlation coefficients: **, p < 0.01, ***, p < 0.001.

## Supplementary Figure 2. Parameter recovery and correlation of the Perseverance model.

1. Parameter recovery of the Perseverance model. The Perseverance model was fitted to the choice behavior that was generated using a Perseverance model with arbitrary chosen free parameters for 1,000 sessions. Correlation coefficients between chosen and fitted parameters were comparable to reported values (Daw et al., 2011), justifying our parameter fitting: α, 0.50; β, 0.38; τ, 0.78; $Q_{0}^{\text{ALP}}$, 0.45, $Q_{0}^{\text{MNP}}$, 0.43, $Q_{0}^{\text{ILP}}$, 0.58, *φ*^ALP^, 0.67; *φ*^MNP^, 0.54; *φ*^ILP^, 0.65.
2. Correlation between estimated parameters in the Perseverance model to assess independence of the parameters. Correlation coefficients were acceptably small (0.0023 to 0.26) implying independence between free parameters.

## Supplementary Figure 3. Mouse brain sections showing DA sensor expression and an optic fiber track.

Coronal brain sections of mice showing expression pattern of the DA sensor, GRAB_DA2m,_ (white area) and the insertion track of the optic fiber targeted at the VS. Animal IDs are indicated at the upper left of each image. The dashed line shows an optical fiber track. Scale bar, 500 µm.

## Supplementary Figure 4. Detection of magazine nosepokes

1. Inside of an operant chamber. Dotted half circle shows the border line for magazine nosepoke detection. Arrows indicate lever-ports (levers are retracted). An arrowhead shows the magazine. Scale bar: 2.5 cm.
2. Mice in an operant chamber. The distance of an animal’s head to the center of the magazine was measured using the reflective tape on the mouse’s head. Left, lever pressing. Middle, timing of a nosepoke detection. Right, putative starting time of magazine checking for a food reward.

## Supplementary Figure 5. Original behavioral data for METH experiments

These panels show original data for Figure 4C. Each dot represents counts of each behavior during a session for PR lever press tasks. Out of 16 mice, one data point is missing for each of three mice, due to malfunctions in the measuring devices or damage to the optical fiber. ALP, active lever press; MNP, magazine nosepoke; ILP, inactive lever press.
