# Supplementary figures and images for: A reinforcement learning model with choice traces for a progressive ratio schedule
Source: Front Behav Neurosci. 2024 Jan 10;17:1302842. doi: 10.3389/fnbeh.2023.1302842 (PMC10806202; doi:10.3389/fnbeh.2023.1302842)

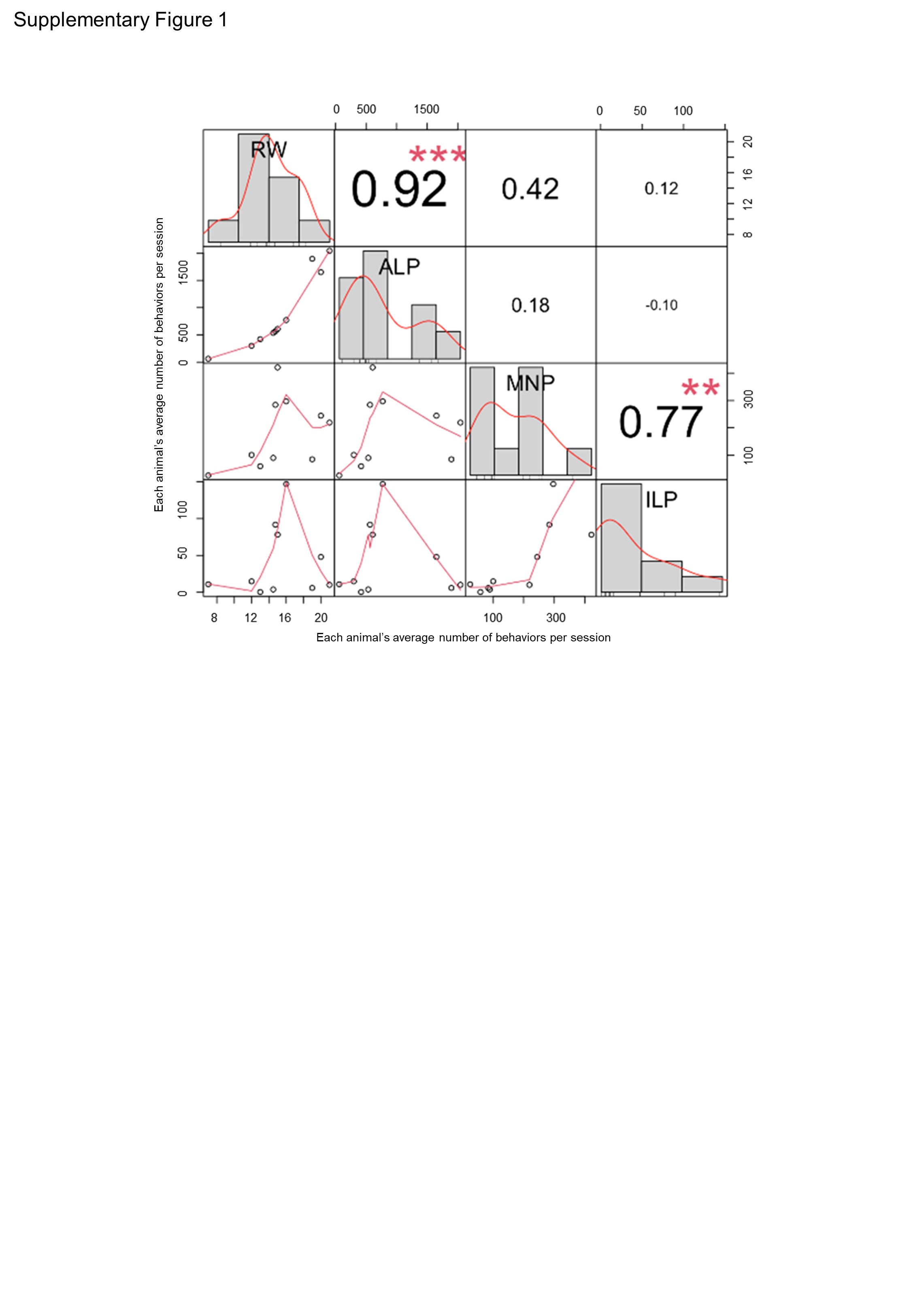

Supplement: Supplementary file 2 [file Image_1.jpg]

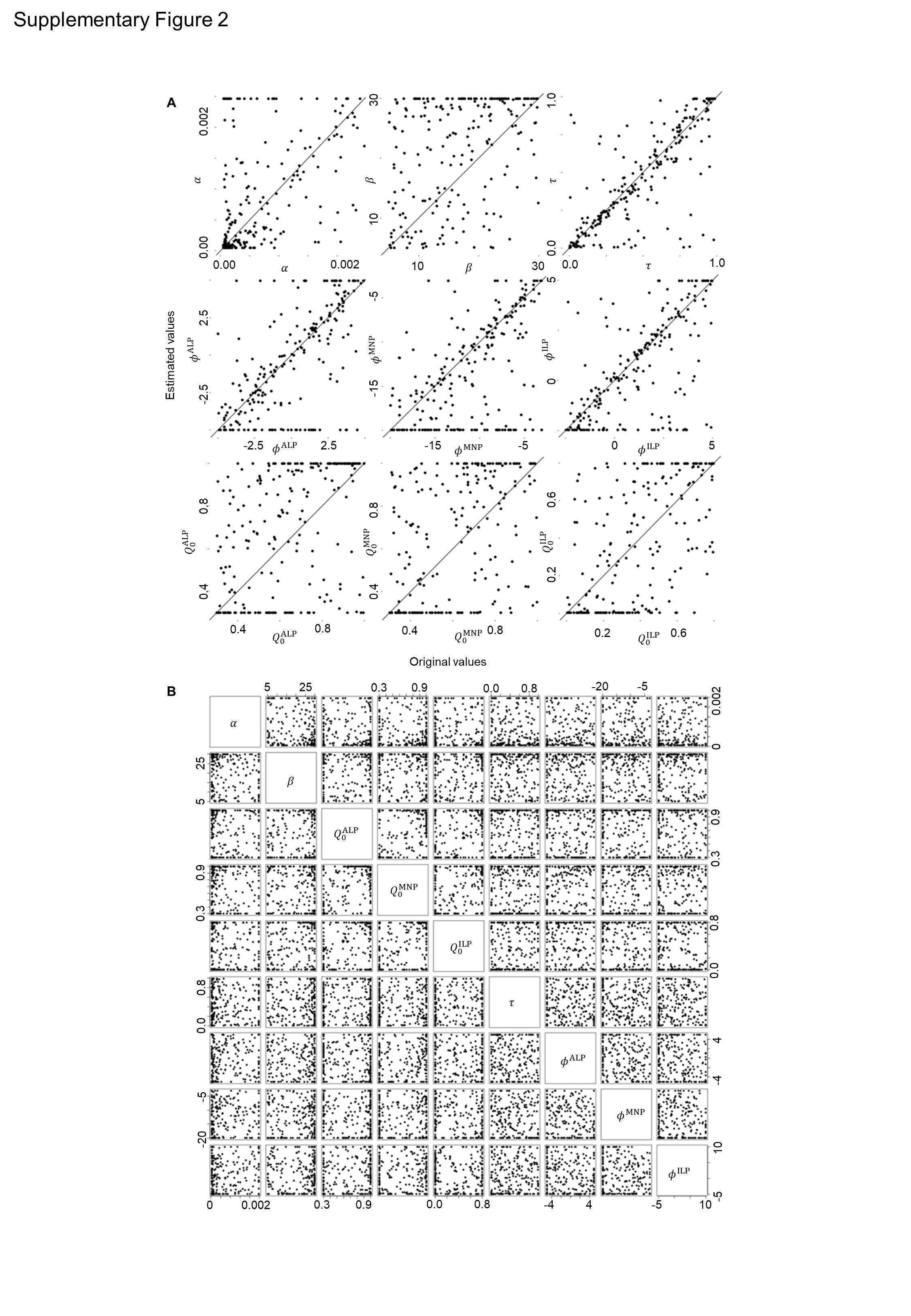

Supplement: Supplementary file 3 [file Image_2.jpg]

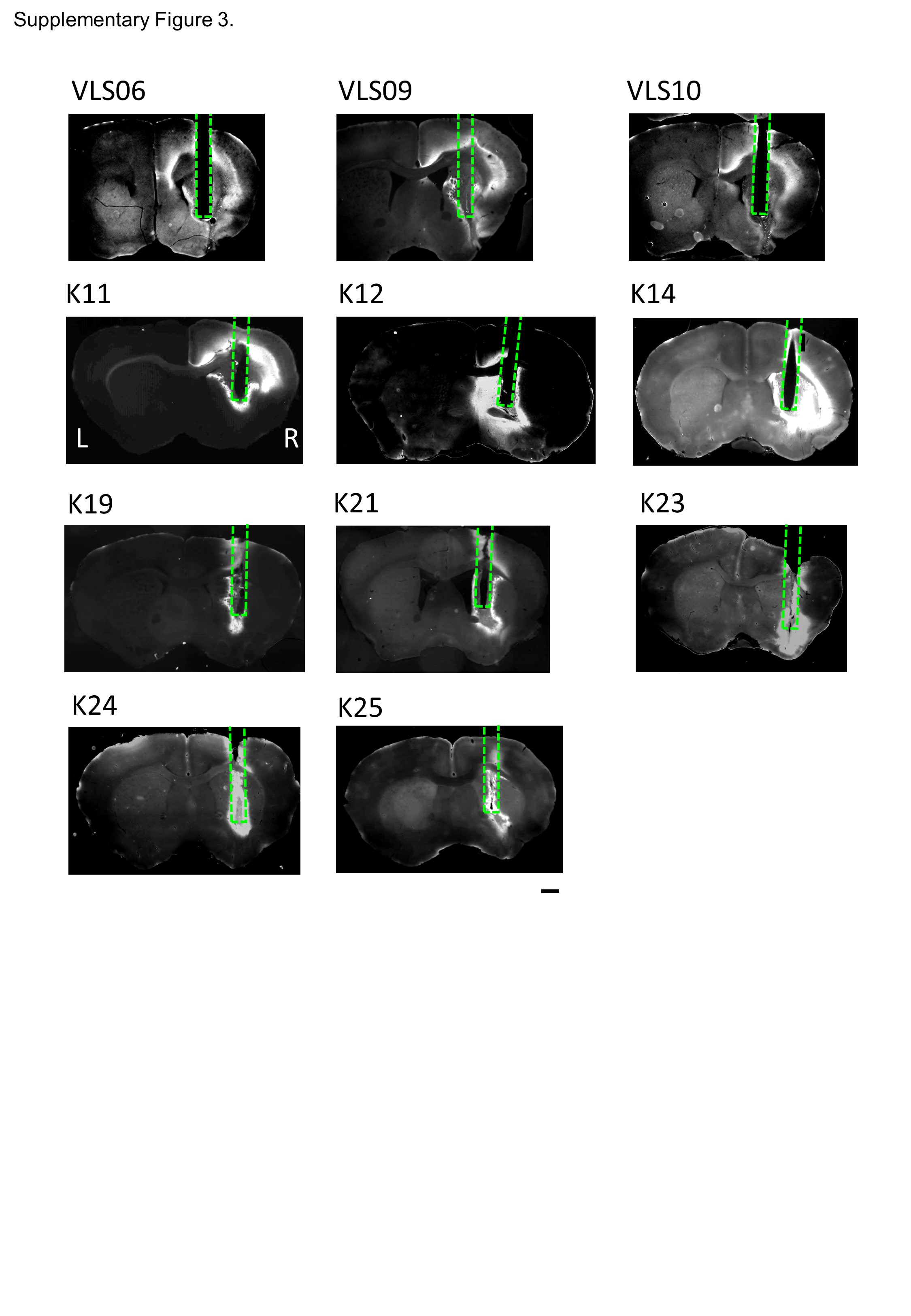

Supplement: Supplementary file 4 [file Image_3.jpg]

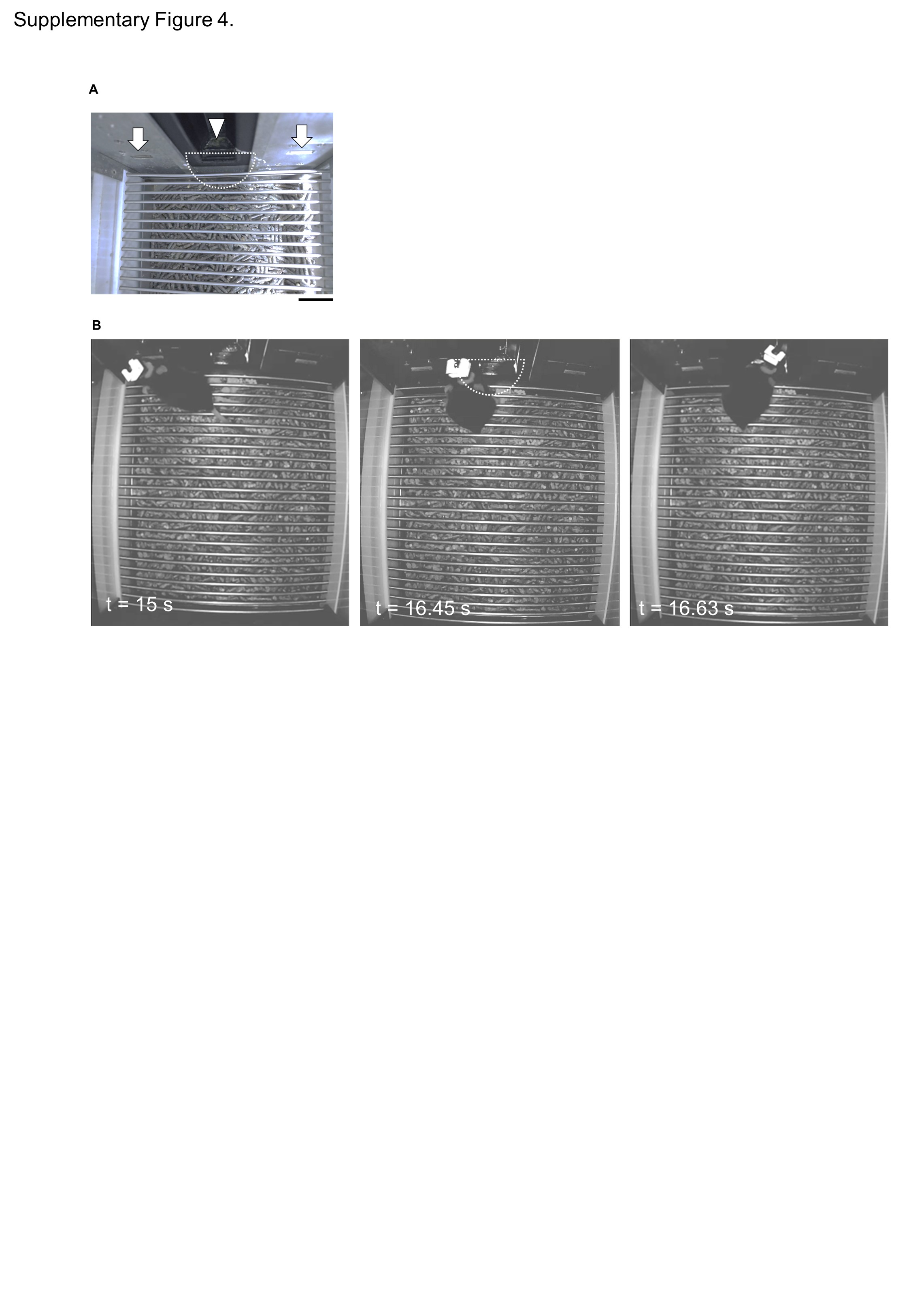

Supplement: Supplementary file 5 [file Image_4.jpg]

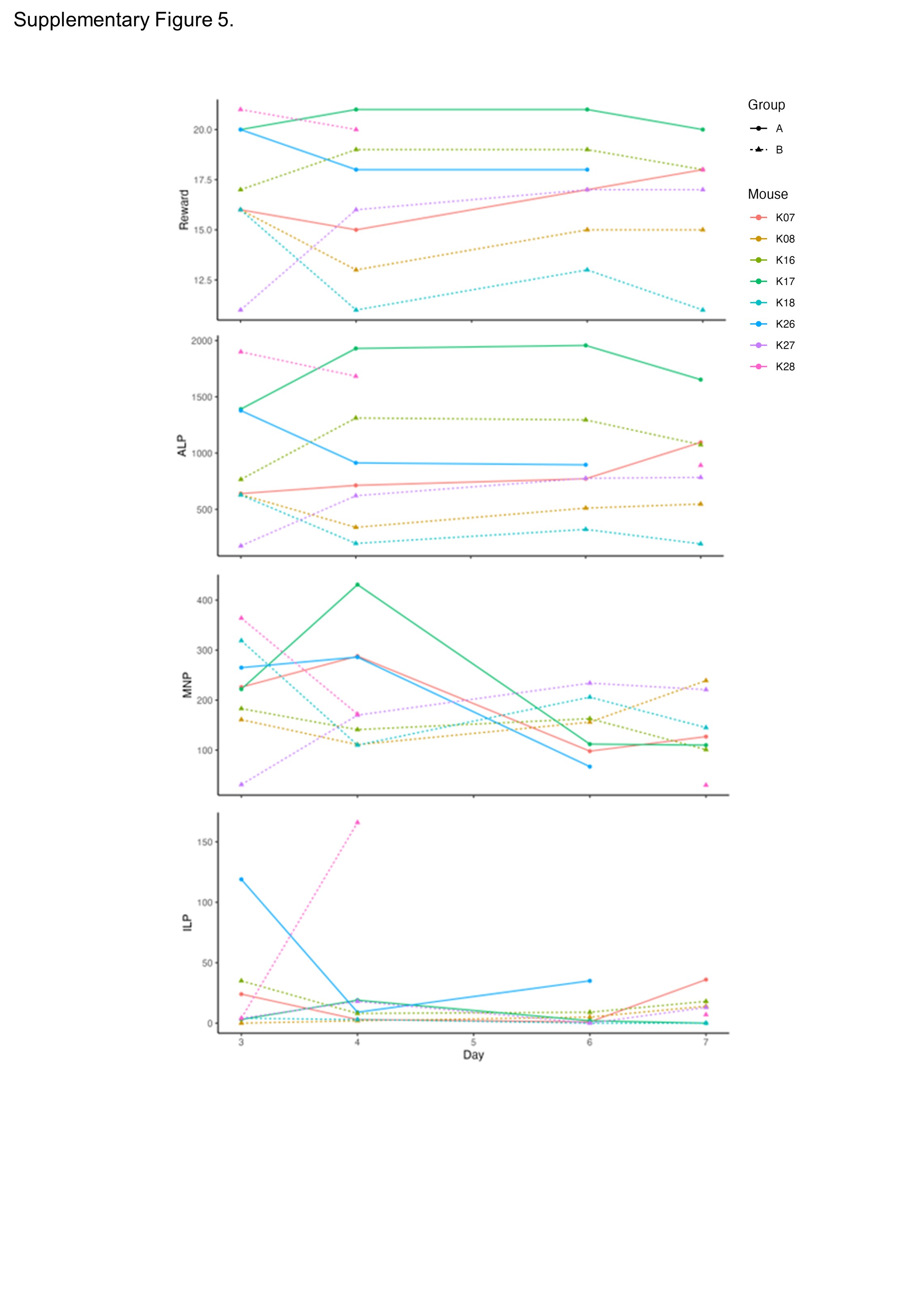

Supplement: Supplementary file 6 [file Image_5.jpg]
